# Effects of Transition from Remote to In-Person Learning in University Students: A Longitudinal Study

**DOI:** 10.3390/ejihpe14060118

**Published:** 2024-06-19

**Authors:** Aline Siteneski, Melina de la Cruz-Velez, Karime Montes-Escobar, Julia Patricia Duran-Ospina, Carolina Fonseca-Restrepo, Mónica Daniela Barreiro-Linzán, Gusdanis Alberto Campos García, Joana Gil-Mohapel

**Affiliations:** 1 School of Medicine, Faculty of Health Sciences, Universidad Técnica de Manabí, Portoviejo 130102, Ecuador; mdelacruz5299@utm.edu.ec (M.d.l.C.-V.);; 2 Research Institute, Universidad Técnica de Manabí, Portoviejo 130105, Ecuador; 3 Department of Mathematics and Statistics, Institute of Basic Sciences, Technical University of Manabí, Portoviejo 130105, Ecuador; karime.montes@gmail.com (K.M.-E.); monica.barreiro@utm.edu.ec (M.D.B.-L.); 4 Statistics Department, University of Salamanca, 37007 Salamanca, Spain; 5 Optometry Carrer, Faculty of Health Sciences, Universidad Técnica de Manabí, Portoviejo 130105, Ecuador; jduran@utm.edu.ec; 6 Departamento de Medicina Veterinaria, Facultad de Ciencias Veterinarias, Universidad Técnica de Manabí, Portoviejo 130105, Ecuador; carolina.fonseca@utm.edu.ec; 7 Island Medical Program, Faculty of Medicine, University of British Columbia, Victoria, BC V8P 5C2, Canada; jgil@uvic.ca; 8 Division of Medical Sciences, University of Victoria, Victoria, BC V8P 5C2, Canada

**Keywords:** anxiety, depression, health sciences students

## Abstract

Previous studies have shown that the transition from the University environment to remote learning impacted student mental health. Our study aimed to investigate the effects of university environment on anxiety and depressive symptoms in health sciences students. Students at the Technical University of Manabí, Ecuador, with 6–10 in-person semesters, who shifted to remote learning and then returned to face-to-face learning were selected. Students responded to the General Anxiety Disorder-7 (GAD-7) and Patient Health Questionnaire-9 (PHQ-9). In addition, questions regarding social interaction, physical exercise, mood and sleep habits were also asked. This longitudinal study tracked 323 students during the return to in-person classes and term end. The results showed similar rates of anxiety (GAD-7, *p* = 0.011-*p* = 0.002) and depression (PHQ-9 *p* = 0.001-*p* = 0.032) among students at week 1 and week 15. Previous diagnosis of depression (OR, 0.171; CI 0.050–0.579, *p* < 0.005) was shown to correlate with depression levels in week 1, with no changes seen at follow-up. Anxiety levels were shown to be associated with a previous diagnosis of the disorder at week 1, but not at follow-up (OR 0.233; CI 0.085–0.643, *p* < 0.005). The return to in-person learning among university students maintained levels of anxiety and depressive symptoms, underscoring ongoing vulnerabilities to mental health disorders in this group.

## 1. Introduction

Mood disorders, including anxiety and major depressive disorder, have a higher prevalence and are associated with increased disability rates when compared to many other diseases [1]. Indeed, depression ranks at 13th and anxiety at 24th among the leading causes of disability-adjusted life-year burden worldwide [1,2], with depression and anxiety being common comorbidities in young adults (18–25 years) [3,4]. Anxiety disorders include a group of mental health conditions characterized by persistent feelings of worry, fear, and real or imaginary apprehension. Anxiety symptoms can include physical manifestations such as palpitations, shortness of breath, and dizziness [5,6]. On the other hand, major depressive disorder is characterized by at least one discrete depressive episode, lasting at least two weeks [4]. Major depressive disorder must involve apparent changes in mood, interests, pleasure, cognition, and symptoms such as sleep and appetite disturbances [7]. Of note, both anxiety and major depressive disorder occur more frequently among women than men [8,9]. Both environmental factors and genetic predisposition are known to influence the development of anxiety and depressive symptoms [10,11].

Among environmental factors, it is worth mentioning the influence of physical activity and sleep quality on mental health. Previous studies report that individuals that engage in higher levels of physical activity had 17% to 21% lower odds of developing depression [12,13]. On the other hand, sleep disorders, mainly insomnia, have been shown to increase the risk of depression and anxiety disorders [13,14,15], while sleep deprivation is also known to impair memory and cognition [14,15]. Of note, poor sleep habits as well as low levels of physical activity occur frequently among university students [16,17,18,19].

Multiple studies conducted in different countries have reported a decline in the mental health of university students during the COVID-19 lockdown [20,21,22,23]. Specifically, it was observed that younger university students were more likely to self-report the occurrence of anxiety, depression and stress during the COVID-19 confinement [24,25]. The shift to remote learning and the challenges associated with adapting to the remote environment have been associated with these findings of poor mental health [26,27,28]. More specifically, a decrease in physical activity and lack of social interaction have been identified as potential risk factors for poor mental health outcomes during the COVID-19 pandemic in young university students [29,30]. Furthermore, suicidal ideation was also shown to increase in university students with a previous diagnosis of psychiatric disorders that were subjected to remote learning during the COVID-19 pandemic [31,32].

University students in the health sciences comprise a population that is considered particularly vulnerable to mental health problems [33]. Depression and anxiety are the most common psychiatric disorders among university students, and these mood disorders appear to be associated with the requirement for high academic standards as well as social aspects related with the university environment [33,34,35]. In the health sciences in particular, meeting high academic standards can be a significant source of stress for students, due to the rigorous demands of these professional programs and engagement in clinical training [33,36]. In agreement, higher anxiety and depression levels have been reported in medical students when compared to the general population [33,37,38]. Moreover, the risk of experiencing depression and anxiety in nursing students is higher when compared to students in other fields of study [36,39].

Our study aimed to investigate the prevalence of anxiety and depression symptoms in students who had previous experience with face-to-face instruction, transitioned to remote learning during the COVID-19 pandemic, and returned to face-to-face instruction after the COVID-19 confinement. Specifically, we have formulated two hypotheses: (1) there will be a notable degree of stability in anxiety and depressive symptoms among health sciences students at the Technical University of Manabí, Ecuador, from the beginning (week 1) to the end (week 15) of the term upon returning to in-person classes; (2) social interaction, physical exercise, and previous diagnoses of mental health disorders will emerge as crucial predictors of anxiety and depressive symptoms among these students during the transition from remote learning to in-person classes. Health science students were available (including Medicine, Clinical Laboratory Sciences, Nursing, Nutrition, and Optometry). To our knowledge, this is the first study that explores the impact of the return to in-person instruction after remote learning on the mental health of university students.

## 2. Materials and Methods

### 2.1. Study Design

We performed a prospective longitudinal observational study with university students in the health sciences at the Technical University of Manabí, Ecuador. Data collection occurred between May 2022 and September 2022 (corresponding to the second semester of 2022 in the academic schedule). The manuscript followed the recommendations of STROBE [40].

### 2.2. Setting

This study was conducted at the Technical University of Manabí, in the Portoviejo coastal region located in Ecuador (South America). The Technical University of Manabí includes a population of approximately 30,220 students, of which 2500 are enrolled in health sciences programs.

### 2.3. Eligibility Criteria and Outcomes

University students in the health sciences (Medicine, Clinical Laboratory Sciences, Nursing, Nutrition, and Optometry) that were older than 18 years of age and enrolled in their sixth or higher semester at the time of recruitment were invited to participate in this study. Student participants had completed one or more semester of in-person classes in a university environment prior to the COVID-19 lockdown. During the COVID-19 confinement, all recruited students had four semesters of remote learning. At the time of the study, participants were enrolled in semester 6–10 of their respective health sciences program. Data were collected at the beginning (week 1) and end (week 15) of the term comprised exclusively of in-person instruction in the university setting. Final analyses were performed using complete data sets from the same participants, collected at the two time-points (weeks 1 and 15) with any incomplete data sets being excluded from the analysis. Worsening of anxiety and depressive symptoms was considered a primary outcome, whereas significant associations between levels of physical exercise and/or sleep quality with worsening of anxiety and depressive symptoms were considered secondary outcomes.

### 2.4. Data Sources/Measurement

Data were collected at two distinct time points, the beginning of in-person classes at the start of the term in the university setting (week 1) and follow-up at the end of the term (i.e., after completion of 15 weeks of in-person instruction in the university setting; week 15). Data were coded, with each participant being given an identification number and no names or personal identifiers being used, in an effort to reduce potential biases in the sample. Statistical analyses were performed using complete data sets, from students who submitted data at both time points (weeks 1 and 15). Incomplete date sets were excluded from the final analysis.

### 2.5. Anxiety and Depression Symptoms of Students

The Patient Health Questionnaire-9 (PHQ-9) was used to assess symptoms of depression in participant health sciences students [41]. Using this validated nine-item questionnaire, participants indicate how often they have been afflicted by each symptom over the past two weeks on a four-point scale. Scores ranging from 5 to 9 suggest mild, 10 to 14 moderate, 15 to 19 moderately severe, and 20 to 27 severe depression. The psychometric properties of the PHQ-9 have a test–retest reliability of 0.84 and internal consistency of 0.89 [42]. The present study used the Spanish version of the PHQ-9 questionnaire (psychometric properties Cronbach’s = 0.89 compared to the original version) [43]. 

The Generalized Anxiety Disorder 7-item Scale (GAD-7) questionnaire [44] was used to evaluate the frequency of anxiety symptoms. Scores ranging from 5 to 9 suggest mild, from 10 to 14 moderate, and greater than 15 severe anxiety symptoms. The scale has shown excellent internal consistency (Cronbach’s = 0.92) and good test–retest reliability (correlation = 0.83). In the present study, the Spanish version [45] of GAD-7 was used (psychometric properties from Cronbach = 0.93, test–retest correlation = 0.92).

### 2.6. Sociodemographic Information of Students

Sociodemographic variables including age, sex, marital status, place of residence, health sciences program and semester enrolled were captured. In addition, data on environmental factors during the COVID-19 confinement and upon return to the in-person university setting were also collected through responses to the following questions: (1) did you have opportunities for in-person social interaction with individuals of your age during the pandemic? (possible answers: 1 corresponds to no; 2 corresponds to yes; 3 corresponds to eventually). (2) Do you engage in at least 150 min (30 min a day, 5 times per week) of physical exercise weekly? (possible answers: Yes or No). (3) Do you feel that the environment (confinement or in-person university setting) influences your mood? (possible answers: Yes or No). (4) How many hours do you sleep per night? (possible answers: I sleep less than 6 h; I sleep between 6 and 8 h; I suffer from insomnia). (5) What time do you usually go to bed? (possible answers: before 10:00 p.m.; between 10:00 p.m. and 12:00 a.m.; after midnight). (6) Have you been previously diagnosed with anxiety or depression and/or are you taking any pharmaceutical therapy for one of these disorders? (possible answers: Yes or No).

### 2.7. Sample Size

The sample size for this study was determined using an online calculator (http://www.raosoft.com/samplesize.html accessed on 5 January 2022). The sample size was calculated based on a population of approximately 2500 university students in health sciences programs at the Technical University of Manabí, Ecuador. It used a confidence interval (CI) of 95% and a margin of error of 5%. The number of complete data sets required for the study was calculated as 334. A total of 520 questionnaires were applied to account for a possible loss of data due to incomplete or unusable questionnaires (estimated at 30%). In the final dataset, the total sample included 323 students who successfully submitted complete data at both time points (week 1 and week 15 of an in-person instruction term).

### 2.8. Ethics Approval

The study was approved by the Human Research Ethics Committee of the Technical University of Manabí, Ecuador (protocol, 2022/CEISH-UTM-INT_17-8). All participants in the study provided written informed consent.

### 2.9. Statistical Analysis

All statistical analyses were performed in SPSS 26 (Armonk, NY, USA: IBM Corp). Sociodemographic data were analyzed using descriptive statistical analysis based on discrete, categorical, or ordinal variables presented in frequencies (N) and percentages (%) (Table 1). The Shapiro–Wilk test was used, and univariate analyses were performed using the Chi-square test (Table 2 and Table 3). A binary logistic regression analysis was performed to identify positive predictors for the presence and/or absence of depression (PHQ-9) and anxiety (GAD-7). The following variables were considered: age, sex, marital status, place of residence, and the six questions included in the surveys administered at Weeks 1 and 15 (see above for details). The odds ratio (OR) was adjusted, and the 95% confidence interval (95% CI) was considered when expressing possible correlations (Table 3). OR were also calculated and a heat map was generated so as to ascertain general trends for the various variables analyzed (*p* < 0.05). The significance level was set at α = 0.05. 

## 3. Results

### 3.1. Results

**Figure 1:**
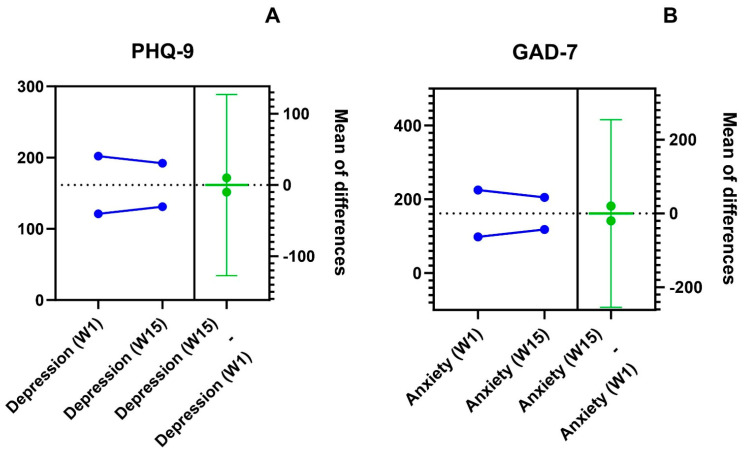
Plots of health outcomes of interest (i.e., (**A**) depression symptoms as assessed with the PHQ-9 tool and (**B**) anxiety symptoms as assessed with the GAD-7 tool), at weeks 1 and 15 of a term of in-person learning.

This study assessed the influence of in-person university learning on anxiety and depressive symptoms in Ecuadorian health science students between March 2022 and September 2022. A total of 520 students were invited to participate in the study and respond to questionnaires at the beginning of week 1 of in-person classes in the university setting (Supplemental Table S1). At follow-up (week 15), 323 students completed the second set of questionnaires. Complete data sets from students that responded to all questionnaires at both week 1 and week 15 were included in the statistical analysis (Figure 1). 

### 3.2. Descriptive Demographic Data

Table 1 summarizes the sociodemographic characteristics of students who completed questionnaires at weeks 1 (beginning of term) and 15 (end of term). The final data set included 323 university students enrolled in health sciences programs, of which 212 students identified as female (65.33%), and 111 identified as male (34.67%). The mean age of the participants was 22.43 years of age. With regard to marital status, most participants (290/323) reported to be single (89.78%) and most lived in urban areas (81.11%).

### 3.3. Main Results

Significant differences were found with regard to self-reported symptoms of anxiety and depression between week 1 (beginning of term) and week 15 (end of term) (Supplementary Table S2). Depression rates (as assessed with the PHQ-9) were significant in females in both week 1 (*p* = 0.001) and week 15 (*p* = 0.032) when compared with males (Table 2). However, Student’s T test revealed no significant differences in PHQ-9 and GAD-7 between 1 and 15 weeks of in-person instruction (*p* > 0.999, Figure 1). Nevertheless, significant differences were seen with regard to the rates of depression among different academic programs at both week 1 (*p* = 0.001) and week 15 (*p* = 0.004). The rates of depression varied between academic programs, being higher in Medicine and lower in Clinical Laboratories. No significant differences among academic programs were observed for other variables studied.

Anxiety rates (as assessed with the GAD-7) were significant in females in both week 1 (*p* = 0.011) and week 15 (*p* = 0.002) when compared with males (Supplementary Table S2). Medicine and Nursing students exhibited higher levels of anxiety when compared to students in other health programs at both week 1 and week 15 (*p* = 0.000 and *p* = 0.004, respectively). No significant differences were observed for other variables at both time points. Depression and anxiety scores remained similar across 15 weeks of in-person learning.

We observed a positive correlation for question #1 (“Did you have opportunities for in-person social interaction with individuals of your age during the pandemic?”) with depression rates at week 1 (*p* = 0.001) and week 15 (*p* = 0.026) and anxiety rates for week 1 (*p* = 0.000) and week 15 (*p* = 0.027). For question #2 (“Do you engage in at least 150 min (30 min a day, five times per week) of physical exercise weekly?”), there was also a positive correlation for depression at week 1 (*p* = 0.000) and week 15 (*p* = 0.010), as well as anxiety at week 1 (*p* = 0.004) and week 15 (*p* = 0.010). When evaluating question #3 (“Do you feel that the environment (confinement or in-person university setting) influences your mood?”), there was no positive correlation with depression rates at week 1 (*p* = 0.099), but significant effects at week 15 (*p* = 0.012), and anxiety at week 1 (*p* = 0.027) and week 15 (*p* = 0.009). For question #4 (“How many hours do you sleep per night?”), there was a positive effect for both depression and anxiety at both time points (*p* = 0.000). Question #5 (“What time do you usually go to bed?”) revealed a positive effect for depression both at week 1 (*p* = 0.000) and week 15 (*p* = 0.002), with going to bed after midnight being significantly associated with depressive symptoms. With regard to anxiety rates, no statistical significance was seen at week 1 (*p* = 0.060), but an increase in anxiety rates was found to be correlated with going to sleep after midnight at week 15 (*p* = 0.001). Finally, question #6 (“Have you been previously diagnosed with anxiety or depression, and/or are you taking any pharmaceutical therapy for one of these disorders?”) revealed significant correlations for both depression rates at week 1 (*p* = 0.000) and week 15 (*p* = 0.004), and anxiety rates at week 1 (*p* = 0.000) and week 15 (*p* = 0.013) (Supplementary Table S3). 

#### 3.3.1. Depressive Symptoms in Students following One and Fifteen Weeks of Exposure to In-Person University Setting

The binomial logistic regression for depression (PHQ-9) was statistically significant at week 1, with a higher risk of experiencing depressive symptoms seen in women (OR 2.276, 95% CI 1.220–4.247, *p* = 0.010). There was no statistical significance at week 15 (*p* = 0.216) (Table 2). For question #1 (“Did you have opportunities for in-person social interaction with individuals of your age during the pandemic?”) (odds ratio [OR], 2.273; 95% confidence interval [CI] 1.105–4.678), the occurrence of depressive symptoms was significantly different for those who responded “No” when compared to those who responded ‘‘Yes” (*p* < 0.026) at week 1, with no statistical differences observed at week 15. This suggests that social interaction during the period of distance learning helped prevent the self-reporting of depressive symptoms at week 1. For question #2 (“Do you engage in at least 150 min (30 min a day, 5 times per week) of physical exercise weekly?”) (odds ratio [OR], 1.966; 95% confidence interval [CI] 1.099–3.516), the occurrence of depressive symptoms was also significantly different for those who responded “No” when compared to those who responded ‘‘Yes” (*p* < 0.023) at week 1. These results imply that physical exercise mitigated depression levels during confinement. In addition, statistically significant differences were also obtained for question #4 (“How many hours do you sleep per night?”) at week 1, with the response “I sleep less than 6 h” being associated with a higher risk of developing depressive symptoms (*p* < 0.001), while the response “I sleep between 6 and 8 h” was associated with a lower risk (odds ratio [OR], 0.336; 95% confidence interval [CI] 0.173–0.653). At week 15, sleeping “between 6 and 8 h” also showed a significant correlation with depressive symptoms (odds ratio [OR], 0.551; 95% confidence interval [CI] 0.324–0.940), *p* < 0.029, highlighting the importance of sleep duration as a protective factor against depressive symptoms. For question #5 (“What time do you usually go to bed?”), a significant difference in the occurrence of depressive symptoms is observed between those who responded “Before 10:00 p.m.” and those who selected “Between 10:00 *p*.m. and 12:00 a.m.” at week 1 (odds ratio [OR], 1.872; 95% confidence interval [CI] 0.999–3.510), *p* < 0.050. No differences were observed at week 15. In week 1, for question #6 (“Have you been previously diagnosed with anxiety or depression and/or are you taking any pharmaceutical therapy for one of these disorders?”), the response “No” (odds ratio [OR], 0.171; 95% confidence interval [CI] 0.050–0.579), *p* < 0.005, is associated with a lower risk of depressive symptoms when compared to the group who responded “Yes”. 

**Figure 2:**
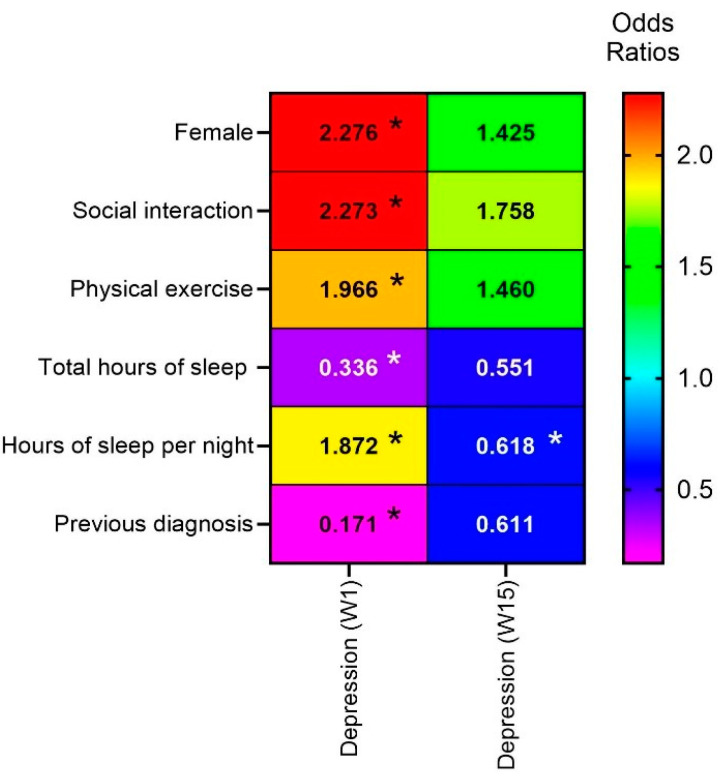
Heat map to compare the odds ratios for depression levels (PHQ-9) weeks 1 and 15 of a term of in-person learning. * (Q1) Did you have social interaction (socialize personally) with individuals of your age during the pandemic? (Q2) Do you do at least 150 min of physical exercise weekly? (Q3) Do you feel that the environment (confinement or university) influences your state of mind? (Q4) How many hours do you sleep per night? (Q5) Do you usually go to bed? (Q6) Are you diagnosed with anxiety or depression and/or are you undergoing drug therapy for one of these disorders?

A heat map was created so as to facilitate visualization of odds ratios associated with depression (PHQ-9), with statistical significance set at *p* < 0.05 (Figure 2). The primary aim was to assess the association between researcher-formulated questions and PHQ-9 scores. The OR for gender decreased from 2.276 to 1.425 over 15 weeks, indicating a reduced association between being female and depressive symptoms across the two time points. The OR for lack of social interaction decreased from 2.273 to 1.758, indicating a reduced correlation between social interaction and depressive symptoms over time. Similarly, the correlation between lack of exercise and depressive symptoms also decreased over time from 1.966 to 1.460. On the other hand, with regard to sleep duration, the OR increased from 0.336 to 0.551, suggesting a slight increase in the association between sleeping 6–8 h per night and a lower probability of developing depressive symptoms. For question #5, the OR decreased from 1.872 to 0.618, indicating a considerable reduction in the relationship between going to bed before 10 p.m. and depressive symptoms. For question #6, an increase in the odds ratio from 0.171 to 0.611 was observed, suggesting an increase in the association between a prior diagnosis and the probability of experiencing depressive symptoms.

#### 3.3.2. Anxiety Symptoms in Students following One and Fifteen Weeks of Exposure to In-Person University Setting

The binomial logistic regression for anxiety (GAD-7) revealed statistically significant results at week 1 for the gender variable (OR 1.886, 95% CI 1.007–3.531). The self-reported GAD-7 scores for women were statistically different from those reported by men (*p* = 0.048). However, the effect of gender was not observed at follow-up (week 15). Significant differences were also detected with regard to question #1 (“Did you have opportunities for in-person social interaction with individuals of your age during the pandemic?”) (OR, 2.294 and 95% CI, 1.182–4.453 for “Eventually”; OR, 3.115 and 95% CI, 1.522–6.373 for “No”), with both answers being positively correlated with self-reported GAD-7 scores at week 1 (*p* < 0.014 and *p* < 0.02, respectively). Regarding question #3 (“Do you feel that the environment (confinement or in-person university setting) influences your mood?”), significant differences were observed in GAD-7 scores at week 1 between those who responded “Yes” and those who responded “No” (*p* = 0.027, OR 0.195, 95% CI 0.046–0.827). With regard to question #4 (“How many hours do you sleep per night?”), the GAD-7 score for those who responded “I sleep less than 6 h” was significantly different from that of those who responded “I sleep between 6 and 8 h” at week 1 (*p* < 0.010 and *p* < 0.04, respectively) OR 0.378, 95% CI, 0.195–0.733. Significant differences were also detected with regard to question #6 (“Have you been previously diagnosed with anxiety or depression and/or are you taking any pharmaceutical therapy for one of these disorders?”) (OR, 0.233; 95% CI, 0.085–0.643), with the response “Yes” being positively correlated with GAD-7 scores at week 1 (*p* < 0.05). No significant differences were seen at week 15. These results suggest a decrease in the correlation between these variables and GAD-7 scores over time. This trend was observed among university health science students following a term of in-person learning.

We used odds ratios to assess correlations between responses to specific questions and anxiety scores in the GAD-7 questionnaire. For the gender variable, there was a slight decrease in odds ratio from 1.886 at week 1 to 1.796 at week 15. For question #1, a significant decrease in odds ratio from 3.115 at week 1 to 1.775 at week 15 was evident, suggesting that over time, the lack of social interaction is less related to anxiety symptoms than in the first week of the term. For question #3, the odds ratio increased slightly from 0.195 at week 1 to 0.418 at week 15. This indicates that, over time, the perception that the environment does not influence mood is more associated with a lower probability of anxiety symptoms. For question #4, a slight decrease in OR from 0.378 at week 1 to 0.508 at week 15 was seen, suggesting that sleeping between 6 and 8 h is slightly less related to a lower probability of anxiety symptoms over time. Finally, for question #6, an increase in the odds ratio from 0.233 at week 1 to 0.387 at week 15 was observed. This indicates that the absence of a prior diagnosis is related to a lower probability of anxiety symptoms in university health science students following a term of in-person learning (Table 3).

**Figure 3:**
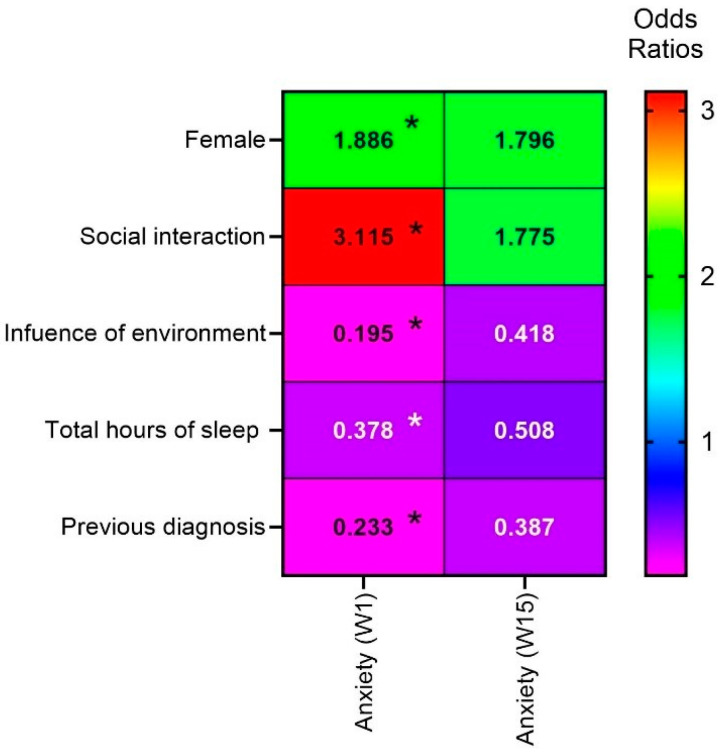
Heat map to compare the odds ratios for anxiety (GAD-7) weeks 1 and 15 of a term of in-person learning. * (Q1) Did you have social interaction (socialize personally) with individuals of your age during the pandemic? (Q2) Do you do at least 150 min of physical exercise weekly? (Q3) Do you feel that the environment (confinement or university) influences your state of mind? (Q4) How many hours do you sleep per night? (Q5) Do you usually go to bed? (Q6) Are you diagnosed with anxiety or depression and/or are you undergoing drug therapy for one of these disorders?

A heat map was created so as to facilitate visualization of odds ratios associated with anxiety (GAD-7), with statistical significance set at *p* < 0.05 (Figure 3).

## 4. Discussion

To the best of our knowledge, this is the first longitudinal study to evaluate the influence of returning to the in-person university environment on affective behaviors in university health science students. Additionally, the potential impacts of social interaction, mood (confinement and university), physical exercise, sleep habits, and previous diagnosis (anxiety or depression) on the levels of depression and anxiety were also assessed. We hypothesized that exposure to an in-person university environment after experiencing distance learning during the COVID-19 confinement had an impact on the levels of depression and anxiety. Our results show that the self-reported levels of depression and anxiety did not improve following 15 weeks of in-person instruction in the university setting when compared to baseline (week 1 of in-person instruction, immediately following the COVID-19 confinement and remote instruction) (*p* > 0.999). Of note, absence of social interaction, level of physical exercise, and previous diagnosis were all shown to be correlated with depression levels at week 1, with no significant changes seen at follow-up (week 15). Overall, these results show that anxiety and depressive symptoms persisted upon the return to in-person learning for university students. The high rates of anxiety and depression scores and their persistence over time indicate that young university students are particularly vulnerable to these mood disorders. 

Several studies conducted in different countries have reported worsening of mental health in university students during the COVID-19 confinement [20,21,22]. In fact, young university students were more likely to self-report depressive symptoms during the COVID-19 confinement [24]. Moreover, academic performance and social isolation have also been shown to impact anxiety levels in university students [32]. Interestingly, in our study, self-reported levels of anxiety and depression symptoms were maintained upon return to the in-person university environment among health sciences students. Indeed, the increased susceptibility of university students to mental disorders was well established before the COVID-19 confinement, and our study corroborates these findings [46,47,48]. 

It is well established that depression increases suicide risk, representing the second leading cause of death among individuals aged 15–29 years [49]. Of note, previous studies have shown increased scores of suicidal ideation associated with mental disorders in college students [47,50]. The suicide prevalence rates between 2011 and 2020 in Ecuador indicate that young adults constitute the most vulnerable population [51]. In addition, a previous study conducted in the same city as the present study showed a prevalence of 8.6% of depressive symptoms among the general population [52]. Furthermore, women are twice as likely as men to suffer from anxiety and depression disorders [9,53,54,55]. Our findings corroborate these studies, since female university students reported the highest rates of anxiety and depression. 

Our study also included a questionnaire with items on the existence of social connections during the COVID-19 confinement, the influence of the university setting on mood, and engagement in physical activity. This questionnaire was administered to students at the beginning of in-person classes (week 1) and then again at the last week of classes, once a full term of in-person learning was completed (week 15). Of note, our student population had already experienced face-to-face classes prior to the COVID-19 pandemic, distance learning during the COVID-19 confinement (4 semesters), followed by return to in-person instruction post-COVID-19 pandemic. Previous studies have indicated that a decrease in social connectivity was strongly associated with increased anxiety and depression symptoms during the COVID-19 confinement [56,57]. In addition, a strong association between low levels of physical activity and poor mental health has been extensively reported in the literature [12,58], with low physical activity levels being correlated with increased anxiety and depressive symptoms in university students [23]. In our findings, both the levels of depression and anxiety were correlated with the lack of social interaction during the COVID-19 confinement among university health sciences students at week 1. On the other hand, the level of physical activity students engaged in also significantly influenced self-reported depressive symptoms at week 1. 

Sleep disturbances, such as insomnia and hypersomnia, have been associated with cognitive impairment and emotional disorders [59,60,61,62]. In addition, poor sleep quality has also been associated with an increased risk of depression [57,58] and is known to contribute to the persistence and severity of depressive symptoms in young adults [63]. Previous studies supporting a strong association between anxiety levels and poor sleep hygiene practices or insomnia among university students [16,17]. A bidirectional relationship between sleep deprivation and anxiety has been previously described, with anxiety disorder causing sleep disturbances, and dysregulation of sleep patterns worsening anxiety symptoms [64]. Indeed, according to the National Sleep Foundation recommendations on sleep hygiene, young adults are advised to sleep between 7 and 9 h per night and maintain the same sleep schedule, so as to optimize their mental and physical well-being. In our study, self-reported sleep habits (specifically 6 to 8 h of sleep per night) did not negativity affect depression or anxiety scores in university health sciences students both at return to face-to-face instruction (week 1) and at follow-up (week 15, following a full term of in-person instruction in the university setting). 

The hypothesis that returning to an in-person university setting after confinement in students with previous experience with face-to-face learning would improve their mental health was not corroborated by our data. Nevertheless, the persistence of anxiety and depression symptoms in students after return to an in-person university setting warrants further investigation in future studies. The majority of participants in our study were aged between 21 and 25. Of note, the prefrontal cortex (known to be involved in impulse control, as well as other higher-order cognitive functions) is the last region of the central nervous system to complete its development, which occurs approximately between 20 and 25 years of age [65], which corresponds to the age range of the majority of the students that participated in our study. Moreover, altered prefrontal cortex activity has been implicated in both depression [66] and anxiety [67]. Thus, future studies are warranted to explore other causes underlying the development of depression and anxiety in young university students, particularly within the health sciences programs.

### Limitations and Strengths of the Study

The main limitation of our study relates to the intrinsic characteristics of a longitudinal study, which simply allows for associations to be made and no definitive cause–effect relationships to be established. Another important limitation of our study was the use of the PHQ-9 and GAD-7 questionnaires to assess levels of depressive and anxiety symptoms among our student population. Indeed, it is important to note that while these two validated questionnaires are commonly used in research studies to assess the occurrence of depression and anxiety symptoms in populations, these are simply screening tools and cannot be used to accurately diagnose these conditions [43,68,69]. Additionally, limitations, including the emergence of new challenges at the end of a university semester, such as exams, fatigue, and internships, can influence students’ responses to PHQ and GAD questionnaires. Of note, students with high PHQ-9 and/or GAD-7 scores were confidentially referred for further diagnosis and psychological/psychiatric treatment at the university student welfare services. Finally, the rigorous health sciences programs that students were enrolled in and their academic performance throughout the 15 weeks of the study are variables that may have also had an impacted on the mental health of participants, particularly at the end of the term (week 15). While in the present study this factor was not included in the analysis, future studies may wish to consider how academic performance may interact with remote versus in-person learning when it comes to the mental health of the student population.

Although previous reports have indicated that students have a preference for face-to-face instruction [70], remote learning has been practiced for a long time. Nevertheless, the COVID-19 pandemic represents the first time that this has been implemented on such a large scale among university students in the health sciences. However, to the best of our knowledge, the effects of returning to in-person instruction following prolonged remote learning on the mental health of this particular student population have never been investigated. As such, one of the strengths of our study was the focus on this specific student population (young university students enrolled in health sciences programs), which has been reported to be particularly susceptible to psychiatric disorders [33,35]. Another strength of this study was its high level of internal and external validity. Indeed, the homogeneous student sample included in this study ensures its positive external validity when compared with the student populations at other universities. Furthermore, our study design attempted to reduce the risk of bias with sample selection, and all study participants had previous experience with in-person learning in the university setting pre-pandemic, distance learning during the COVID-19 confinement, and a return to face-to-face instruction post-pandemic (being followed during their first term after return to in-person instruction in the university setting).

## 5. Conclusions

Our results showed persistence in self-reported symptoms of anxiety and depression in university students enrolled in health sciences programs who experienced remote learning during the COVID-19 confinement, following one term (15 weeks) of in-person instruction. The increased vulnerability of this student population to mental health disorders points to the importance of developing potential programs and policies aimed at promoting wellbeing in the university setting. In addition, future studies are warranted to determine how face-to-face learning itself and/or social interactions in an in-person university setting impact the mental health of university students who experienced remote learning during the COVID-19 pandemic.

## Figures and Tables

**Table 1:** Sociodemographic data of participants included in the study (who completed all questionnaires both at the start and at the end of the 15-week period of in-person learning in the university setting).

Sociodemographic Background	Weeks 1 and 15 of In-Person Learning
	(N = 323)
Age groups	
<20	32 (9.91%)
21–25	267 (82.66%)
26–30	18 (5.57%)
31+ years	6 (1.86%)
Sex	
Female	212 (65.33%)
Male	111 (34.67%)
Marital status	
Married	15 (4.64%)
Unmarried	290 (89.78%)
Divorced	3 (0.93%)
Free Union	15 (4.64%)
Living location	
Rural	61 (18.89%)
Urban	262 (81.11%)
Career	
Nutrition and Dietetics	42 (13%)
Optometry	48 (14.86%)
Medicine	129 (39.94%)
Nursing	19 (5.88%)
Clinical Laboratory	85 (26.32%)

Note: N, frequency; %, percentage.

**Table 2:** Logistic regression analysis for prediction of self-reported depressive symptoms (PHQ-9 scores).

Sociodemographic Background	First Week of Class (PHQ-9)				Fifteenth Week of Class (PHQ-9)			
	*p*-Value	Odds Ratios	95% CI		*p*-Value	Odds Ratios	95% CI	
			Lower	Upper			Lower	Upper
Age groups								
<20 (Reference)	0.371							
21–25	0.082	0.437	0.175	1.102	0.811	0.85	0.220	3.262
26–30	0.581	0.605	0.093	3.805	0.403	2.36	0.321	17.274
31+ years	1.000	0.000	0.000		0.857	0.41	0.205	54.866
Sex								
Male								
Female	**0.010**	**2.276**	**1.220**	**4.247**	0.216	1.425	0.813	2.497
Marital status								
Free Union (Reference)	0.942				0.886			
Married	0.688	1.602	0.160	16.046	0.501	0.550	0.096	3.138
Divorced	0.554	2.878	0.087	95.708	0.747	0.602	0.027	13.202
Unmarried	0.633	1.580	0.243	10.287	0.891	0.918	0.273	3.087
Living location								
Urban (Reference)								
Rural	0.590	0.783	0.321	1.907	0.857	0.931	0.427	2.028
Career								
Optometry (Reference)	0.480				0.238			
Nursing	0.718	0.773	0.190	3.138	0.763	0.822	0.231	2.931
Clinical Laboratory	0.153	0.485	0.180	1.310	0.268	0.610	0.254	1.463
Medicine	0.830	0.914	0.401	2.080	0.603	1.220	0.577	2.580
Nutrition	0.295	0.571	0.200	1.631	0.232	0.549	0.205	1.469
Q1								
Yes (Reference)	**0.003**				0.213			
Eventually	0.167	1.594	0.823	3.088	0.226	1.424	0.804	2.522
No	**0.026**	**2.273**	**1.105**	**4.678**	0.097	1.758	0.903	3.420
Q2								
Yes (Reference)								
No	**0.023**	**1.966**	**1.099**	**3.516**	0.167	1.460	0.853	2.497
Q3								
Yes (Reference)								
No	0.357	0.559	0.162	1.926	0.081	0.402	0.145	1.120
Q4								
Less than 6 h (Reference)	**0.001**				**0.018**			
Between 6 and 8 h	**0.001**	**0.336**	**0.173**	**0.653**	**0.029**	**0.551**	**0.324**	**0.940**
Insomnia	0.301	1.952	0.550	6.937	0.128	3.653	0.690	19.333
Q5								
Between 10:00 p.m. and 12:00 a.m. (Reference)	0.148				0.403			
Before 10:00 p.m.	**0.050**	**1.872**	**0.999**	**3.510**	0.328	0.618	0.235	1.622
After 12:00 a.m.	1.000	0.000	0.000		0.179	1.457	0.841	2.525
Q6								
Yes (Reference)								
No	**0.005**	**0.171**	**0.050**	**0.579**	0.314	0.611	0.235	1.593

Note: OR = odds ratio; 95% CIs = 95% confidence intervals; statistically significant effects (*p* < 0.05) are in bold. (Q1) Did you have social interaction (socialize personally) with individuals of your age during the pandemic? (Q2) Do you engage in at least 150 min of physical exercise weekly? (Q3) Do you feel that the environment (confinement or university) influences your state of mind? (Q4) How many hours do you sleep per night? (Q5) At what time do you usually go to bed? (Q6) Are you diagnosed with anxiety or depression and/or are you undergoing drug therapy for one of these disorders?

**Table 3:** Logistic regression analysis for prediction of self-reported anxiety symptoms (GAD-7 scores).

Sociodemographic Background	First Week of Class (GAD-7)				Fifteenth Week of Class (GAD-7)			
	*p*-Value	Odds Ratios	95% CI		*p*-Value	Odds Ratios	95% CI	
			Lower	Upper			Lower	Upper
Age groups								
<20 (Reference)	0.730							
21–25	1.000	0.441	0.000		1.000	0.000	0.000	
26–30	0.515	31.663	0.000		1.000	1.191	0.132	11.332
31+ years	0.263	14.533	0.000		1.000	2.080	0.117	39.745
Sex								
Male								
Female	**0.048**	**1.886**	**1.007**	**3.531**	0.054	1.796	0.991	3.254
Marital status								
Free Union (Reference)	0.918				0.967			
Married	0.882	1.183	0.129	10.854	0.620	1.564	0.267	9.148
Divorced	0.496	3.427	0.099	118.731	0.999	0.000	0.000	
Unmarried	0.887	1.133	0.202	6.334	0.811	1.171	0.321	4.270
Living location								
Urban (Reference)								
Rural	0.208	0.557	0.224	1.384	0,489	0.746	0.326	1.707
Career								
Optometry (Reference)	0.052				0.233			
Nursing	0.472	1.647	0.423	6.405	0.383	1.812	0.476	6.894
Clinical Laboratory	0.203	0.517	0.187	1.428	0.840	.907	0.354	2.326
Medicine	0.469	1.347	0.602	3.014	0.148	1.815	0.809	4.068
Nutrition	0.155	0.459	0.157	1.343	0.916	0.946	0.339	2.643
Q1								
Yes (Reference)	**0.005**				0.226			
Eventually	**0.014**	**2.294**	**1.182**	**4.453**	0.811	1.076	0.591	1.957
No	**0.002**	**3.115**	**1.522**	**6.373**	0.097	1.775	0.901	3.498
Q2								
Yes (Reference)								
No	0.504	1.218	0.683	2.170	0.533	1.196	0.681	2.099
Q3								
Yes (Reference)								
No	**0.027**	**0.195**	**0.046**	**0.827**	0.121	0.418	0.139	1.258
Q4								
Less than 6 h (Reference)	**0.010**				0.006			
Between 6 and 8 h	**0.004**	**0.378**	**0.195**	**0.733**	0.016	0.508	0.292	0.883
Insomnia	0.949	0.966	0.339	2.754	0.061	5.778	0.920	36.297
Q5								
Between 10:00 p.m. and 12:00 a.m. (Reference)	0.581				0.261			
Before 10:00 p.m.	0.298	0.707	0.368	1.358	0.090	0.431	0.163	1.140
After 12:00 a.m.	1.000	0.000	0.000		0.125	1.555	0.884	2.736
Q6								
Yes (Reference)								
No	**0.005**	**0.233**	**0.085**	**0.643**	0.058	0.387	0.145	1.032

Note: OR = odds ratio; 95% CIs = 95% confidence intervals; statistically significant effects (*p* < 0.05) are in bold. (Q1) Did you have social interaction (socialize personally) with individuals of your age during the pandemic? (Q2) Do you engage in at least 150 min of physical exercise weekly? (Q3) Do you feel that the environment (confinement or university) influences your state of mind? (Q4) How many hours do you sleep per night? (Q5) At what time do you usually go to bed? (Q6) Are you diagnosed with anxiety or depression and/or are you undergoing drug therapy for one of these disorders?

## Data Availability

Data are contained within the article. Additional data are available from the corresponding author upon request.

## Supplementary Materials

The following supporting information can be downloaded at https://www.mdpi.com/article/10.3390/ejihpe14060118/s1: Table S1: Sociodemographic characteristics of the participants who completed questionnaires at the start of the term (week 1 of in-person learning) and at the end of the term (week 15 on in-person learning). Table S2. PHQ-9 and GAD-7 responses of participants included in the study (who completed all questionnaires both at the start and at the end of the 15-week period of in-person learning in the university setting) organized by sociodemographic background. Table S3. PHQ-9 and GAD-7 responses of participants included in the study (who completed all questionnaires both at the start and at the end of the 15-week period of in-person learning in the university setting) organized according to the responses obtained to questions 1–6.

## References

[1] Ferrari A.J., Santomauro D.F., Herrera A.M.M., Shadid J., Ashbaugh C., Erskine H.E., Charlson F.J., Degenhardt L., Scott J.G., McGrath J.J. (2022). Global, regional, and national burden of 12 mental disorders in 204 countries and territories, 1990–2019: A systematic analysis for the Global Burden of Disease Study 2019. Lancet Psychiatry.

[2] Vos T., Barber R., Bell B., Bertozzi-Villa A., Biryukov S., Bolliger I., Charlson F., Davis A., Degenhardt L., Dicker D. (2015). Global, regional, and national incidence, prevalence, and years lived with disability for 301 acute and chronic diseases and injuries in 188 countries, 1990–2013: A systematic analysis for the Global Burden of Disease Study 2013. Lancet.

[3] Kessler R., Berglund P., Demler O., Jin R., Merikangas K., Walters E. (2005). Lifetime Prevalence and Age-of-Onset Distributions of DSM-IV Disorders in the National Comorbidity Survey Replication. Arch. Gen. Psychiatry.

[4] American Psychiatric Association (2014). Desk Reference to the Diagnostic Criteria from DSM-5.

[5] Szuhany K., Simon N. (2022). Anxiety Disorders: A Review. JAMA.

[6] Shevlin M., Hyland P., Nolan E., Owczarek M., Ben-Ezra M., Karatzias T. (2022). ICD-11 ‘mixed depressive and anxiety disorder’ is clinical rather than sub-clinical and more common than anxiety and depression in the general population. Br. J. Clin. Psychol.

[7] Otte C., Gold S.M., Penninx B.W., Pariante C.M., Etkin A., Fava M., Mohr D.C., Schatzberg A.F. (2016). Major depressive disorder. Nat. Rev. Dis. Prim..

[8] Penninx B., Pine D., Holmes E., Reif A. (2021). Anxiety disorders. Lancet.

[9] Kuehner C. (2017). Why is depression more common among women than among men?. Lancet Psychiatry.

[10] Hettema J., Prescott C., Myers J., Neale M., Kendler K. (2005). The Structure of Genetic and Environmental Risk Factors for Anxiety Disorders in Men and Women. Arch. Gen. Psychiatry.

[11] Lopizzo N., Chiavetto L.B., Cattane N., Plazzotta G., Tarazi F.I., Pariante C.M., Riva M.A., Cattaneo A. (2015). Gene-Environment Interaction in Major Depression: Focus on Experience-Dependent Biological Systems. Front. Psychiatry.

[12] Schuch F.B., Vancampfort D., Firth J., Rosenbaum S., Ward P.B., Silva E.S., Hallgren M., De Leon A.P., Dunn A.L., Deslandes A.C. (2018). Physical Activity and Incident Depression: A Meta-Analysis of Prospective Cohort Studies. Am. J. Psychiatry.

[13] Dishman R., McDowell C., Herring M. (2021). Customary physical activity and odds of depression: A systematic review and meta-analysis of 111 prospective cohort studies. Br. J. Sports Med..

[14] Ford D., Kamerow D. (1989). Epidemiologic Study of Sleep Disturbances and Psychiatric Disorders An Opportunity for Prevention?. JAMA.

[15] Li L., Wu C., Gan Y., Qu X., Lu Z. (2016). Insomnia and the risk of depression: A meta-analysis of prospective cohort studies. BMC Psychiatry.

[16] Manzar D., Noohu M.M., Salahuddin M., Nureye D., Albougami A., Spence D.W., Pandi-Perumal S.R., BaHammam A.S. (2020). Insomnia Symptoms and Their Association with Anxiety and Poor Sleep Hygiene Practices among Ethiopian University Students. Nat. Sci. Sleep.

[17] Anwer S., Alghadir A., Manzar M., Noohu M., Salahuddin M., Li H. (2019). Psychometric Analysis of the Sleep Hygiene Index and Correlation with Stress and Anxiety among Saudi University Students. Nat. Sci. Sleep.

[18] Guthold R., Stevens G., Riley L., Bull F. (2018). Worldwide trends in insufficient physical activity from 2001 to 2016: A pooled analysis of 358 population-based surveys with 1.9 million participants. Lancet Glob. Health.

[19] Ghrouz A.K., Noohu M.M., Manzar D., Spence D.W., BaHammam A.S., Pandi-Perumal S.R. (2019). Physical activity and sleep quality in relation to mental health among college students. Sleep Breath.

[20] Giusti L., Mammarella S., Salza A., Del Vecchio S., Ussorio D., Casacchia M., Roncone R. (2021). Predictors of academic performance during the COVID-19 outbreak: Impact of distance education on mental health, social cognition and memory abilities in an Italian university student sample. BMC Psychol..

[21] Seetan K., Al-Zubi M., Rubbai Y., Athamneh M., Khamees A., Radaideh T. (2021). Impact of COVID-19 on medical students’ mental wellbeing in Jordan. PLoS ONE.

[22] Di Consiglio M., Merola S., Pascucci T., Violani C., Couyoumdjian A. (2021). The Impact of COVID-19 Pandemic on Italian University Students’ Mental Health: Changes across the Waves. Int. J. Environ. Res. Public Health.

[23] Li B., Tong W.-X., Zhang M., Wang G.-X., Zhang Y.-S., Meng S.-Q., Li Y.-X., Cui Z.-L., Zhang J.-Y., Ye Y.-P. (2022). Epidemiological Study of Physical Activity, Negative Moods, and Their Correlations among College Students. Int. J. Environ. Res. Public Health.

[24] Ochnik D., Rogowska A., Kuśnierz C., Jakubiak M., Schütz A., Held M., Arzenšek A., Benatov J., Berger R., Korchagina E.V. (2021). Mental health prevalence and predictors among university students in nine countries during the COVID-19 pandemic: A cross-national study. Sci. Rep..

[25] Chatterjee S., Barikar M., Mukherjee A. (2020). Impact of COVID-19 pandemic on pre-existing mental health problems. Asian J. Psychiatr..

[26] Husky M., Kovess V., Swendsen J. (2020). Stress and anxiety among university students in France during COVID-19 mandatory confinement. Compr. Psychiatry.

[27] Patsali M.E., Mousa D.-P.V., Papadopoulou E.V., Papadopoulou K.K., Kaparounaki C.K., Diakogiannis I., Fountoulakis K.N. (2020). University students’ changes in mental health status and determinants of behavior during the COVID-19 lockdown in Greece. Psychiatry Res..

[28] Lopes A., Nihei O. (2021). Depression, anxiety and stress symptoms in Brazilian university students during the COVID-19 pandemic: Predictors and association with life satisfaction, psychological well-being and coping strategies. PLoS ONE.

[29] Ammar A., Mueller P., Trabelsi K., Chtourou H., Boukhris O., Masmoudi L., Bouaziz B., Brach M., Schmicker M., Bentlage E. (2020). Psychological consequences of COVID-19 home confinement: The ECLB-COVID19 multicenter study. PLoS ONE.

[30] Fitzpatrick K.M., Harris C., Drawve G. (2020). Fear of COVID-19 and the mental health consequences in America. Psychol. Trauma.

[31] Li W., Yin J., Cai X., Cheng X., Wang Y. (2020). Association between sleep duration and quality and depressive symptoms among university students: A cross-sectional study. PLoS ONE.

[32] Cao W., Fang Z., Hou G., Han M., Xu X., Dong J., Zheng J. (2020). The psychological impact of the COVID-19 epidemic on college students in China. Psychiatry Res..

[33] Rotenstein L.S., Ramos M.A., Torre M., Segal J.B., Peluso M.J., Guille C., Sen S., Mata D.A. (2016). Prevalence of Depression, Depressive Symptoms, and Suicidal Ideation among Medical Students: A Systematic Review and Meta-Analysis. JAMA.

[34] Barbayannis G., Bandari M., Zheng X., Baquerizo H., Pecor K., Ming X. (2022). Academic Stress and Mental Well-Being in College Students: Correlations, Affected Groups, and COVID-19. Front. Psychol..

[35] Maharaj S., Lee T., Lal S. (2018). Prevalence and Risk Factors of Depression, Anxiety, and Stress in a Cohort of Australian Nurses. Int. J. Environ. Res. Public Health.

[36] Shaban I., Khater W., Akhu L. (2012). Undergraduate nursing students’ stress sources and coping behaviours during their initial period of clinical training: A Jordanian perspective. Nurse Educ. Pract..

[37] Moutinho I.L.D., Maddalena N.d.C.P., Roland R.K., Lucchetti A.L.G., Tibiriçá S.H.C., Ezequiel O.d.S., Lucchetti G. (2017). Depression, stress and anxiety in medical students: A cross-sectional comparison between students from different semesters. Rev. Assoc. Med. Bras..

[38] Lavergne J., Kennedy M. (2021). Telepsychiatry and Medical Students: A Promising Mental Health Treatment for Medical Student Use Both Personally and Professionally. Curr. Psychiatry Rep..

[39] Bartlett M., Taylor H., Nelson J. (2016). Comparison of Mental Health Characteristics and Stress Between Baccalaureate Nursing Students and Non-Nursing Students. J. Nurs. Educ..

[40] Vandenbroucke J.P., von Elm E., Altman D.G., Gøtzsche P.C., Mulrow C.D., Pocock S.J., Poole C., Schlesselman J.J., Egger M., STROBE Initiative (2014). Strengthening the Reporting of Observational Studies in Epidemiology (STROBE): Explanation and elaboration. Int. J. Surg..

[41] Kroenke K., Spitzer R., Williams J. (2001). The PHQ-9: Validity of a brief depression severity measure. J. Gen. Intern. Med..

[42] Saldivia S., Aslan J., Cova F., Vicente B., Inostroza C., Rincón P. (2019). Propiedades psicométricas del PHQ-9 (Patient Health Questionnaire) en centros de atención primaria de Chile. Rev. Méd. Chile.

[43] Muñoz-Navarro R., Cano-Vindel A., Medrano L.A., Schmitz F., Ruiz-Rodríguez P., Abellán-Maeso C., Font-Payeras M.A., Hermosilla-Pasamar A.M. (2017). Utility of the PHQ-9 to identify major depressive disorder in adult patients in Spanish primary care centres. BMC Psychiatry.

[44] Spitzer R., Kroenke K., Williams J., Löwe B. (2006). A Brief Measure for Assessing Generalized Anxiety Disorder: The GAD-7. Arch. Intern. Med..

[45] Garcia-Campayo J., Zamorano E., Ruiz M.A., Pardo A., Perez-Paramo M., Lopez-Gomez V., Freire O., Rejas J. (2010). Cultural adaptation into Spanish of the generalized anxiety disorder-7 (GAD-7) scale as a screening tool. Health Qual. Life Outcomes.

[46] Auerbach R.P., Alonso J., Axinn W.G., Cuijpers P., Ebert D.D., Green J.G., Hwang I., Kessler R.C., Liu H., Mortier P. (2016). Mental disorders among college students in the World Health Organization World Mental Health Surveys. Psychol. Med..

[47] Auerbach R.P., Mortier P., Bruffaerts R., Alonso J., Benjet C., Cuijpers P., Demyttenaere K., Ebert D.D., Green J.G., Hasking P. (2019). Mental disorder comorbidity and suicidal thoughts and behaviors in the World Health Organization World Mental Health Surveys International College Student initiative. Int. J. Methods Psychiatr. Res..

[48] Alonso J., Vilagut G., Mortier P., Auerbach R.P., Bruffaerts R., Cuijpers P., Demyttenaere K., Ebert D.D., Ennis E., Gutiérrez-García R.A. (2019). The role impairment associated with mental disorder risk profiles in the WHO World Mental Health International College Student Initiative. Int. J. Methods Psychiatr. Res..

[49] World Health Organization (2020). Depression [Internet]. WHO. https://www.who.int/es/news-room/fact-sheets/detail/suicide.

[50] Bruffaerts R., Mortier P., Auerbach R.P., Alonso J., De la Torre A.E.H., Cuijpers P., Demyttenaere K., Ebert D.D., Green J.G., Hasking P. (2019). Lifetime and 12-month treatment for mental disorders and suicidal thoughts and behaviors among first year college students. Int. J. Methods Psychiatr. Res..

[51] Lapo G., Talledo J., Fernández L. (2023). A competing risk survival analysis of the sociodemographic factors of COVID-19 in-hospital mortality in Ecuador. Cad. Saúde Pública.

[52] Montalvo J., Perero M., Portalanza D., Camargo A., Siteneski A. (2021). Prevalence Of Major Depressive Disorder In Portoviejo, Ecuador. Rev. Ecuat. Neurol..

[53] Albert P. (2015). Why is depression more prevalent in women?. J. Psychiatry Neurosci..

[54] Talarowska M., Rucka K., Kowalczyk M., Chodkiewicz J., Kowalczyk E., Karbownik M.S., Sienkiewicz M. (2023). Mental Health of Students at Polish Universities after Two Years of the Outbreak of COVID-19. Int. J. Environ. Res. Public Health.

[55] Gavurova B., Ivankova V., Rigelsky M., Mudarri T., Miovsky M. (2022). Somatic Symptoms, Anxiety, and Depression among College Students in the Czech Republic and Slovakia: A Cross-Sectional Study. Front. Public Health.

[56] World Health Organization (2021). COVID-19 Pandemic Triggers 25% Increase in Prevalence of Anxiety and Depression Worldwide [Internet]. WHO. https://www.who.int/news/item/02-03-2022-covid-19-pandemic-triggers-25-increase-in-prevalence-of-anxiety-and-depression-worldwide.

[57] Pfefferbaum B., North C.S. (2020). Mental Health and the COVID-19 Pandemic. N. Engl. J. Med..

[58] Stubbs B., Vancampfort D., Rosenbaum S., Firth J., Cosco T., Veronese N., Salum G.A., Schuch F.B. (2017). An examination of the anxiolytic effects of exercise for people with anxiety and stress-related disorders: A meta-analysis. Psychiatry Res..

[59] Hirshkowitz M., Whiton K., Albert S.M., Alessi C., Bruni O., DonCarlos L., Hazen N., Herman J., Adams Hillard P.J., Katz E.S. (2015). National Sleep Foundation’s updated sleep duration recommendations: Final report. Sleep Health.

[60] Lowe C., Safati A., Hall P. (2017). The neurocognitive consequences of sleep restriction: A meta-analytic review. Neurosci. Biobehav. Rev..

[61] Krause A.J., Ben Simon E., Mander B.A., Greer S.M., Saletin J.M., Goldstein-Piekarski A.N., Walker M.P. (2017). The sleep-deprived human brain. Nat. Rev. Neurosci..

[62] Yoo S., Hu P., Gujar N., Jolesz F., Walker M. (2007). A deficit in the ability to form new human memories without sleep. Nat. Neurosci..

[63] Baglioni C., Battagliese G., Feige B., Spiegelhalder K., Nissen C., Voderholzer U., Lombardo C., Riemann D. (2011). Insomnia as a predictor of depression: A meta-analytic evaluation of longitudinal epidemiological studies. J. Affect. Disord..

[64] Oh C., Kim H., Na H., Cho K., Chu M. (2019). The Effect of Anxiety and Depression on Sleep Quality of Individuals With High Risk for Insomnia: A Population-Based Study. Front. Neurol..

[65] Gogtay N., Giedd J.N., Lusk L., Hayashi K.M., Greenstein D., Vaituzis A.C., Nugent T.F., Herman D.H., Clasen L.S., Toga A.W. (2004). Dynamic mapping of human cortical development during childhood through early adulthood. Proc. Natl. Acad. Sci. USA.

[66] Hare B., Duman R. (2020). Prefrontal cortex circuits in depression and anxiety: Contribution of discrete neuronal populations and target regions. Mol. Psychiatry.

[67] Park J., Moghaddam B. (2017). Impact of anxiety on prefrontal cortex encoding of cognitive flexibility. Neuroscience.

[68] Alghadir A., Manzar M., Anwer S., Albougami A., Salahuddin M. (2020). Psychometric Properties of the Generalized Anxiety Disorder Scale among Saudi University Male Students. Neuropsychiatr. Dis. Treat..

[69] Manzar D., Alghadir A.H., Anwer S., Alqahtani M., Salahuddin M., Addo H.A., Jifar W.W., Alasmee N.A. (2021). Psychometric Properties of the General Anxiety Disorders-7 Scale Using Categorical Data Methods: A Study in a Sample of University Attending Ethiopian Young Adults. Neuropsychiatr. Dis. Treat..

[70] Atwa H., Shehata M.H., Al-Ansari A., Kumar A., Jaradat A., Ahmed J., Deifalla A. (2022). Online, Face-to-Face, or Blended Learning? Faculty and Medical Students’ Perceptions During the COVID-19 Pandemic: A Mixed-Method Study. Front. Med..

