# Supplementary material for: Effects of Transition from Remote to In-Person Learning in University Students: A Longitudinal Study
Source: Eur J Investig Health Psychol Educ. 2024 Jun 19;14(6):1786–802. doi: 10.3390/ejihpe14060118 (PMC11202480; doi:10.3390/ejihpe14060118)
Supplement: Supplementary file 1 [file ejihpe-14-00118-s001.zip › ejihpe-3029413-supplementary.pdf]

Supplementary Table

Table S1: Sociodemographic characteristics of the participants who completed questionnaires at the start of the term (week 1 of in-person learning) and at the end of the term (week 15 on in-person learning).

**Sociodemographic Background**	**Week 1 of in-person classes (N=520)**	**Week 15 of in-person classes (N=323)**
**Age groups**		
< 20	10 (1.92%)	17 (5.26%)
21 - 25	463 (89.04%)	283 (87.62%)
26 - 30	33 (6.35%)	17 (5.26%)
31 + years	14 (2.69%)	6 (1.86%)
**Sex**		
Female	338 (65%)	212 (65.63%)
Male	182 (35%)	111 (34.37%)
**Marital status**		
Married	23 (4.42%)	13 (4.02%)
Unmarried	460 (88.46%)	295 (91.33%)
Divorced	5 (0.96%)	3 (0.93%)
Free Union	32 (6.15%)	12 (3.72%)
**Living location**		
Rural	120 (23.08%)	71 (21.98%)
Urban	400 (76.92%)	252 (78.02%)
**Semester**		
6	191 (36.73%)	131 (40.56%)
7	200 (38.46%)	113 (34.98%)
8	86 (16.54%)	70 (21.67%)
9	25 (4.81%)	8 (2.48%)
10	18 (3.46%)	1 (0.31%)
**Major**		
Nursing	76 (14.62%)	19 (5.88%)
Clinical laboratory	138 (26.54%)	87 (26.93%)
Medicine	184 (35.38%)	129 (39.94%)
Nutrition and diet	63 (12.12%)	40 (12.38%)
Optometry	59 (11.35%)	48 (14.86%)
**Questions**		
**Q1**		
Yes	235 (45.19%)	145 (44.89%)
No	110 (21.15%)	68 (21.05%)
Eventually	175 (33.65%)	110 (34.06%)
**Q2**		
Yes	259 (49.81%)	132 (40.87%)

No	261 (50.19%)	191 (59.13%)
**Q3**		
Yes	466 (89.62%)	292 (90.40%)
No	54 (10.38%)	31 (9.60%)
**Q4**		
Less than 6 hours	143 (27.50)	135 (41.80)
Between 6 and 8 hours	339 (65.19%)	175 (54.18%)
Insomnia	38 (7.31%)	13 (4.02%)
**Q5**		
After 12:00 a.m.	45 (8.65%)	152 (47.06%)
Before 10:00 p.m.	227 (43.65%)	16 (4.95%)
Between 10:00 p.m. and 12:00 a.m.	248 (47.69%)	155 (47.99%)
**Q6**		
Yes	45 (8.65%)	28 (8.67%)
No	475 (91.35%)	295 (91.33%)

Table S2. PHQ-9 and GAD-7 responses of participants included in the study (who completed all questionnaires both at the start and at the end of the 15-week period of in-person learning in the university setting) organized by sociodemographic background.

**Sociodemographic Background**	**Week 1 of in-person classes (PHQ-9) n (323)**		**Week 15 of in-person classes (PHQ-9) n (323)**	
	**NO (0)**	**YES (1)**	**NO (0)**	**YES (1)**
**Age groups**	p=0.188		p=0.505	
< 20	18 (56,3%%)	14 (43,8%%)	16 (50.0%)	16 (50.0%)
21 – 25	165 (61,8%)	102 (38,2%)	163 (61.0%)	104 (39.0%)
26 – 30	13 (72,2%)	5 (27,8%)	9 (50.0%)	9 (50.0%)
31 + years	6 (100,0%)	0 (0,0%)	4 (66,7%)	2 (33,3%)
**Sex**	**p=0.001**		**p=0.032**	
Female	118 (55,9%)	93 (44,1%)	116 (55,0%)	95 (45,0%)
Male	84 (75,0%)	28 (25,0%)	76 (67,9%)	36 (32,1%)
**Marital status**	p=0.517		p=0.778	
Free Union	12 (80,0%)	3 (20,0%)	9 (60,0%)	6 (40.0%)
Married	10 (66,7%)	5 (33,3%)	11 (73.3%)	4 (26.7%)
Divorced	2 (66.7%)	1 (33.3%)	2 (66.7%)	1 (33.3%)
Unmarried	178 (61,4%)	112 (38,6%)	170 (58,6%)	120 (41,4%)
**Living location**	p=0.186		p=0.471	

Urban	159 (60,7%)	103 (39,3%)	153 (58,4%)	109 (41,6%)
Rural	43 (70,5%)	18 (29,5%)	39 (63,9%)	22 (36,1%)
**Career**	**p=0.001**		**p=0.004**	
Optometry	28 (58,3%)	20 (41,7%)	29 (60,4%)	19 (39,6%)
Nursing	11 (57,9%)	8 (42,1%)	11 (57,9%)	8 (42,1%)
Clinical Laboratory	67 (78,8%)	18 (21,2%)	60 (70,6%)	25 (29,4%)
Medicine	66 (51,2%)	63 (48,8%)	61 (47,3%)	68 (52,7%)
Nutrition	30 (71,4%)	12 (28,6%)	31 (73,8%)	11 (26,2%)

**Sociodemographic Background**	**Week 1 of in-person classes (GAD-7) n (323)**		**Week 15 of in-person classes (GAD-7) n (323)**	
	**NO (0)**	**YES (1)**	**NO (0)**	**YES (1)**
**Age groups**	p=0.432		p=0.386	
< 20	21 (65,6%)	11 (34,4%)	17 (53,1%)	15 (46,9%)
21 – 25	186 (69,7%)	81 (30,3%)	170 (63,7%)	97 (36,3%)
26 – 30	12 (66,7%)	6 (33,3%)	14 (77,8%)	4 (22,2%)
31 + years	6 (100,0%)	0 (0.0%)	4 (50.0%)	2 (50.0%)
**Sex**	**p=0.011**		**p=0.002**	
Female	137 (64,9%)	74 (35,1%)	121 (57,3%)	90 (42,7%)
Male	88 (78,6%)	24 (21,4%)	84 (75,0%)	28 (25,0%)
**Marital status**	p=0.914		p=0.761	
Free Union	12 (80.0%)	3 (20.0%)	10 (66,7%)	5 (33,3%)
Married	11 (73,3%)	4 (26,7%)	10 (66,7%)	5 (33,3%)
Divorced	2 (66,7%)	1 (33,3%)	3 (100,0%)	0 (0,0%)
Unmarried	200 (69,0%)	90 (31,0%)	182 (62,8%)	108 (37,2%)
**Living location**	p=0.216		p=0.239	
Urban	178 (67,9%)	84 (32,1%)	162 (61,8%)	100 (38,2%)
Rural	47 (77,0%)	14 (23,0%)	43 (70,5%)	18 (29,5%)
**Career**	**p=0.000**		**p=0.004**	
Optometry	31 (64,6%)	17 (35,4%)	34 (70,8%)	14 (29,2%)
Nursing	11 (57,9%)	8 (42,1%)	11 (57,9%)	8 (42,1%)
Clinical Laboratory	73 (85,9%)	12 (14,1%)	64 (75,3%)	21 (24,7%)
Medicine	76 (58,9%)	53 (41,1%)	66 (51,2%)	63 (48,8%)
Nutrition	34 (81,0%)	8 (19,0%)	30 (71,4%)	12 (28,6%)

Note: n, frequency; %, percentage

P-values were calculated with the $\chi 2$ test or the Fisher's exact for comparing categorical variables.

*Fisher's exact test was used if >20 % of the cells had expected frequencies of less than five.

Table S3. PHQ-9 and GAD-7 responses of participants included in the study (who completed all questionnaires both at the start and at the end of the 15-week period of in-person learning in the university setting) organized according to the responses obtained to Questions 1-6.

**Questions**	**Week 1 of in-person classes (PHQ-9) n (323)**		**Week 15 of in-person classes (PHQ-9) n (323)**	
	**NO (0)**	**YES (1)**	**NO (0)**	**YES (1)**
**Q1**	**p=0.001**		**p=0.026**	
Yes	103 (74,1%)	36 (25,9%)	97 (66,9%)	48 (33,1%)
No	37 (51,4%)	35 (48,6%)	33 (48,5%)	35 (51,5%)
Eventually	62 (55,4%)	50 (44,6%)	62 (56,4%)	48 (43,6%)
**Q2**	**p=0.000**		**p=0.004**	
Yes	117 (74,1%)	41(25,9%)	91 (68,9%)	41 (31,1%)
No	85 (51,5%)	80 (48,5%)	101 (52,9%)	90 (47,1%)
**Q3**	p=0.099		**p=0.012**	
Yes	181 (61,1%)	115 (38,9%)	167 (57,2%)	125 (42,8%)
No	21 (77,8%)	6 (22,2%)	25 (80,6%)	6 (19,4%)
**Q4**	**p=0.000**		**p=0.000**	
Less than 6 hours	32 (41,0%)	46 (59,0%)	65 (48,1%)	70 (51,9%)
Between 6 and 8 hours	166 (75,1%)	55 (24,9%)	125 (71,4%%)	50 (28,6%)
Insomnia	4 (16,7%)	20 (83,3%)	2 (15,4%)	11 (84,6%)
**Q5**	**p=0.000**		**p=0.002**	
After 12:00 a.m	1 (100,0%)	0 (0,0%)	75 (49,3%)	77 (50,7%)
Before 10:00 p.m.	63 (46,3%)	73 (53,7%)	10 (62,5%)	6 (37,5%)
Between 10:00 p.m. and 12:00 a.m.	138 (74,2%)	48 (25,8%)	107 (69,0%)	48 (31,0%)
**Q6**	**p=0.000**		**p=0.004**	
Yes	4 (15,4%)	22 (84,6%)	9 (32,1%)	19 (67,9%)
No	198 (66,7%)	99 (33,3%)	183 (62,0%)	112 (38,0%)

**Questions**	**Week 1 of in-person classes (GAD-7) n (323)**		**Week 15 of in-person classes (GAD-7) n (323)**	
	**NO (0)**	**YES (1)**	**NO (0)**	**YES (1)**
**Q1**	**p=0.000**		**p=0.027**	
Yes	113 (81,3%)	26 (18,7%)	100 (69,0%)	45 (31,0%)
No	42 (58,3%)	30 (41,7%)	34 (50,0%)	34 (50,0%)
Eventually	70 (62,5%)	42 (37,5%)	71 (64,5%)	39 (35,5%)
**Q2**	**p=0.008**		**p=0.010**	
Yes	121 (76,6%)	37 (23,4%)	95 (72,0%)	37 (28,0%)

No	104 (63,0%)	61 (37,0%)	110 (57,6%)	81 (42,4%)
**Q3**	**p=0.027**		**p=0.009**	
Yes	201 (67,9%)	95 (32,1%)	179 (61,3%)	113 (38,7%)
No	24 (88,9%)	3 (11,1%)	26 (83,9%)	5 (16,1%)
**Q4**	**p=0.000**		**p=0.000**	
Less than 6 hours	41 (52,6%)	37 (47,4%)	70 (51,9%)	65 (48,1%)
Between 6 and 8 hours	173 (78,3%)	48 (21,7%)	133 (76,0%)	42 (24,0%)
Insomnia	11 (45,8%)	13 (54,2%)	2 (15,4%)	11 (84,6%)
**Q5**	p=0.060		**p=0.001**	
After 12:00 a.m	1 (100,0%)	0 (0,0%)	80 (52,6%)	72 (47,4%)
Before 10:00 p.m.	86 (63,2%)	50 (36,8%)	12 (75,0%)	4 (25,0%)
Between 10:00 p.m. and 12:00 a.m.	138 (74,2%)	48 (25,8%)	113 (72,9%)	42 (27,1%)
**Q6**	**p=0.000**		**p=0.013**	
Yes	8 (30,8%)	18 (69,2%)	8 (28,6%)	20 (71,4%)
No	217 (73,1%)	80 (26,9%)	197 (66,8%)	98 (33,2%)

Note: n, frequency; %, percentage

P-values were calculated with the $\chi 2$ test or the Fisher's exact for comparing categorical variables.

*Fisher's exact test was used if >20 % of the cells had expected frequencies of less than five.

*Q1) Did you have social interaction (socialize personally) with individuals of your age during the pandemic? Q2) Do you engage in at least 150 min of physical exercise weekly? Q3) Do you feel that the environment (confinement or university) influences your state of mind? Q4) How many hours do you sleep per night? Q5) At what time do you usually go to bed? Q6) Are you diagnosed with anxiety or depression and/or are you undergoing drug therapy for one of these disorders?
